# HSATII RNA-dependent triplex formation in early human embryogenesis as a potential mechanism for Y chromosome loss in Turner syndrome

**DOI:** 10.3389/fmolb.2025.1731956

**Published:** 2025-11-27

**Authors:** Krystian Fularski

**Affiliations:** Faculty of Medicine, Jagiellonian University Medical College, Kraków, Poland

**Keywords:** HSATII, RNA-DNA triplex, loss of Y chromosome, early human embryogenesis, Turner syndrome

## Abstract

Turner syndrome (TS) arises from partial or complete loss of a sex chromosome, yet the mechanistic basis for Y chromosome loss (LoY), which may contribute to a subset of TS cases, remains unclear. This article addresses the existing gap in knowledge by proposing a hypothesis linking a transient physiological window of elevated HSATII RNA levels during preimplantation embryogenesis to recent bioinformatic predictions indicating that Y-linked HSATII arrays possess uniquely high triplex-forming propensity. In this context, HSATII-derived RNAs could form RNA-DNA triplexes in early embryogenesis preferentially at Y-linked HSATII tracts. If unresolved, these structures may stall replication forks and promote Y chromosome instability which may ultimately lead to complete or partial LoY. The proposed model reframes part of the TS etiology from a stochastic segregation error toward a definable process, and motivates experimental validation of its predictions. If supported by experimental evidence, this framework could further guide the search for modifying factors - such as interindividual variation in the Y-linked HSATII sequence or triplex-resolution efficiency - and, in the longer term, enable risk stratification for Y chromosome instability in potential embryos based on parental molecular profiles. In a broader context, the hypothesis underscores pericentromeric satellite biology as a potentially underexplored contributor to genome stability in early human development.

## Graphical Abstract

**Figure:**
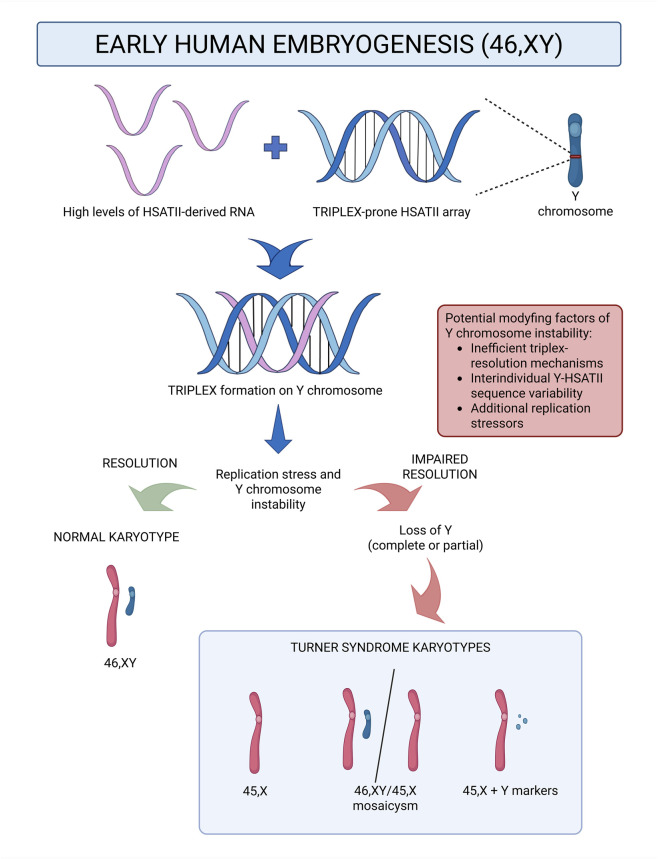

## Introduction

Turner syndrome (TS) is a chromosomal disorder resulting from complete or partial loss of one sex chromosome, most frequently leading to a 45,X karyotype, although other variants such as mosaicism or partial deletions are also observed (Khan et al., 2024). This chromosomal loss is generally attributed to errors such as chromosome lagging or nondisjunction, which can occur either during meiosis in gametogenesis or during early embryonic mitoses, and are typically regarded as stochastic segregation errors (Alves, 2022). While TS can result from the loss of either X or Y chromosome, this work focuses on the loss of Y chromosome (LoY), for which the precise mechanisms remain obscure (Sarel-Gallily et al., 2025). A contributing factor that has been proposed is the absence of CENP-B binding sites on the Y chromosome, which may weaken centromere function (Fachinetti et al., 2015; Ly et al., 2017), although this alone may not fully explain the vulnerability of the Y chromosome to loss in early human embryogenesis.

Notably, the Y chromosome - like several other chromosomes in the human genome - harbors tracts of HSATII (Human Satellite II), a family of pericentromeric DNA tandem repeats, usually embedded in constitutive heterochromatin and considered non-coding (Miga, 2019). Although HSATII is predominantly silent in differentiated cells, low-level transcription has been observed in certain contexts, which is consistent with broader observations that centromeric and pericentromeric repeats are not absolutely inert (Eymery and Callanan, 2009; Enukashvily and Ponomartsev, 2013). More pronounced exceptions are well documented under pathological or stress conditions, including cancer, aging, and other forms of cellular stress (Smurova and De Wulf, 2018; Fonseca-Carvalho et al., 2024). In these contexts, HSATII transcription is carried out by RNA polymerase II, but the precise promoter architecture and the mechanisms governing transcriptional activation of HSATII arrays remain largely unresolved. Available evidence suggests that activation likely reflects a combination of epigenetic derepression and transcription-factor recruitment. In particular, DNA hypomethylation at pericentromeric regions has been proposed to reduce heterochromatin compaction and thereby permit transcription of otherwise silent satellite sequences, including HSATII (Hall et al., 2017). Another available research showed that some proteins potentiate HSATII transcription in specific contexts, including the human factor DUX4 in an ATM-dependent manner during DNA-damage signaling (Shadle et al., 2019), as well as the viral immediate-early proteins, IE1 and IE2 during human cytomegalovirus infection (Nogalski et al., 2019).

When transcriptionally active, HSATII DNA gives rise to abundant long non-coding RNAs (lncRNAs), which accumulate in the nucleus and can affect chromatin architecture (Chan et al., 2017). Importantly, among physiological states, early human development is uniquely characterized by HSATII RNA upregulation, with unusually high levels observed during oocyte maturation and throughout the preimplantation stages, before sharply decreasing by the blastocyst stage (Yandım and Karakülah, 2019; Dobrynin et al., 2020). These RNAs arise from HSATII DNA arrays, which despite high overall similarity, display sequence heterogeneity and variation in copy number across different chromosomes and individuals (Altemose et al., 2022), features that may influence their structural behavior.

HSATII DNA, like other repetitive elements, can adopt non-B DNA conformations and engage in higher-order interactions with nucleic acids. Among these, *triplexes* represent one notable configuration, formed when a third strand of either DNA or RNA complementary to a polypurine or polypyrimidine tract of double helical DNA, binds via Hoogsteen or reverse-Hoogsteen hydrogen bonds to generate a triple helix (Leisegang et al., 2024). Recent bioinformatic analyses predicted that HSATII arrays on the Y chromosome exhibit exceptionally high propensity to form RNA-DNA triplex structures compared with HSATII arrays on other chromosomes. Using the PATO triplex prediction algorithm (Amatria-Barral et al., 2023) the Y chromosome was assigned a score of 3616, which is more than five times greater than that of chromosome 16 (the second most triplex-prone) and over 200-fold greater than that of chromosome 1, which carried the lowest score among all evaluated HSATII-bearing chromosomes (Hegyi and Lexa, 2024). Triplex structures are known to be replication stress-inducing elements, that can cause replication fork stalling (Wells, 2007; Vasquez and Wang, 2013). This phenomenon could be even more pronounced due to the potential presence of other non-B DNA structures that may form at HSATII DNA, such as hairpins or slipped-strand structures (Thakur et al., 2021; Mellor et al., 2022). If unresolved by replication-restart or repair mechanisms before mitosis, replication fork stalling can result in under-replicated DNA (urDNA), which during mitosis may manifest as ultrafine bridges (UFBs) or bulky chromosome bridges - structures associated with nondisjunction, micronuclei formation, and chromosome fragmentation (Siri et al., 2021; Mellor et al., 2022).

Taken together, these bioinformatic predictions indicate that HSATII arrays on the Y chromosome have an exceptionally high propensity for triplex formation compared with HSATII sequences elsewhere in the genome, whereas early embryogenesis represents a unique developmental window marked by abundant HSATII RNA. Furthermore, the mechanisms underlying LoY, which is among the possible cytogenetic causes of TS, remain especially poorly understood.

To date, the potential connection between Y-linked triplex-forming propensity and elevated HSATII RNA levels during early human embryogenesis has not been directly examined. Investigating this relationship could provide a framework for advancing our understanding of the molecular etiology of Turner syndrome cases attributable to Y chromosome loss. This presently uncharacterized intersection forms the basis of the hypothesis presented below.

## Presentation of the hypothesis

The transient upregulation of HSATII RNA that prevails throughout the preimplantation stages of early embryogenesis creates a molecular window in which cells are unusually abundant in HSATII-derived long non-coding RNAs (lncRNAs). These RNAs have the potential to engage in RNA-DNA triplex formation by binding complementary HSATII DNA tracts via Hoogsteen or reverse-Hoogsteen base pairing. Because bioinformatic predictions indicate that HSATII arrays on the Y chromosome have an exceptionally high triplex-forming propensity, this effect would be expected to be particularly pronounced on the Y chromosome. Triplex structures that form on the Y chromosome, if not efficiently resolved by cellular helicases and repair pathways, may stall replication forks during DNA synthesis, generating replication stress. This could result in under-replicated DNA, predisposing to ultrafine bridges (UFBs) or bulky chromosome bridges occurrence during mitosis. These structures are known to cause missegregation or micronucleation events, potentially leading to partial or complete loss of the Y chromosome.

Within this framework, different cytogenetic outcomes of Turner syndrome could arise:

1. Complete Y chromosome loss during the first zygotic division, resulting in a uniformly 45,X karyotype;
2. Partial loss or fragmentation of Y chromosome material during the first zygotic division, which would give rise to marker Y variants;
3. Complete Y chromosome loss after the first division but before blastocyst formation, during the HSATII RNA-rich window, which would cause only a subset of embryonic cells to be Y-depleted resulting in mosaicism (45,X/46,XY).

In cytogenetic surveys, pure 45,X cases together with mosaics involving 45,X/46,XY and Y-marker variants often account for more than 50% of TS karyotypes (Jacobs et al., 1997; Sybert and McCauley, 2004; Cameron-Pimblett et al., 2017, summarized in Ibarra-Ramírez et al., 2023). While many 45,X cases are likely attributable to X chromosome loss, the true proportion of Turner syndrome cases that arise from complete loss of the X versus loss of the Y chromosome is currently unknown, likely because complete early Y loss would have left no detectable Y-derived sequences in the resulting 45,X karyotype, thereby making the distinction difficult to resolve. Despite this uncertainty, the molecular basis of Y chromosome loss remains especially underexplored, and the mechanism proposed here is intended to apply specifically to the subset of TS cases originating from Y chromosome loss.

This leads to the hypothesis that early embryonic HSATII RNA abundance, combined with the predicted exceptional triplex-forming propensity of Y-linked HSATII arrays, may contribute to a notable fraction of TS cases.

### Limitations of the hypothesis

The proposed hypothesis provides a possible framework for understanding Y chromosome loss in a subset of TS cases. However, several limitations must be acknowledged, ranging from its scope of applicability to the current lack of experimental validation.

First, the model presented here focuses specifically on Y chromosome loss and therefore does not account for TS cases arising from X chromosome loss. While addressing the X-loss scenario was beyond the scope of this work, it is important to emphasize that a comprehensive understanding of TS etiopathology will ultimately require integrating mechanisms for both X and Y chromosome abnormalities.

Second, even if this hypothesis proves correct, it does not by itself explain why Y chromosome loss occurs only in a minority of early embryonic cases. TS is observed in approximately 1 in 2,500 live female births (Ibarra-Ramírez et al., 2023) whereas HSATII RNA upregulation and the potential for triplex formation are features of early human embryos in general. This discrepancy highlights the need to identify modifying factors that may determine whether the proposed mechanism translates into chromosomal loss. These may include the previously proposed lack of CENP-B binding sites on the Y chromosome (Fachinetti et al., 2015; Ly et al., 2017), as well as additional possibilities such as interindividual variability in triplex resolution mechanisms, DNA repair capacity, or HSATII sequence and structural variation, including potential differences in HSATII RNA availability for DNA binding. These possible variables are further elaborated in the *Discussion* section below. Rather than diminishing the relevance of the hypothesis, this gap underscores its value as a framework for guiding targeted experimental studies.

A further limitation of this hypothesis lies in its reliance on bioinformatic predictions of Y-linked HSATII triplex propensity. The inference that HSATII arrays on the Y chromosome are exceptionally triplex-prone derives from computational analyses (Hegyi and Lexa, 2024) using the PATO algorithm (Amatria-Barral et al., 2023), which identifies sequence motifs compatible with triplex formation. PATO is grounded in established biochemical principles of Hoogsteen and reverse-Hoogsteen pairing, and its performance was benchmarked by its authors against experimentally validated RNA-DNA triplexes, using the framework introduced by Antonov et al. (Antonov et al., 2019). In these comparisons, PATO outperformed earlier tools such as Triplexator (Buske et al., 2012), demonstrating improved predictive accuracy for known triplex-forming regions. Nevertheless, the genome-wide predictions of the aforementioned PATO algorithm remain theoretical and have not yet been systematically validated *in vitro* or *in vivo*. A related limitation concerns the chromatin-interaction evidence that served as the template for the PATO analysis conducted by Hegyi and Lexa, which consisted of Hi-C data from a single colon cancer cell line (HCT116) (Hegyi and Lexa, 2024). Although both cancer cells and early human embryonic cells exhibit marked HSATII upregulation, their epigenetic landscapes and pericentromeric architectures may differ, as chromatin organization can vary across cell types (Rao et al., 2014; Kim et al., 2020). Therefore, the specific Y-linked HSATII interaction patterns observed in cancer-derived Hi-C datasets may not fully reflect those present during preimplantation development. Nevertheless, many higher-order chromatin features, including large-scale compartmentalization and architectural principles, show broad conservation across diverse cell types (Rao et al., 2014), which provides some support for cautiously extrapolating these observations. Taken together, both basing on colon cancer derived Hi-C data and lack of laboratorial conformation of triplex formation propensity are notable limitations of proposed hypothesis. Although the bioinformatic predictions offer the strongest computational rationale currently available, direct experimental confirmation of triplex formation at Y-linked HSATII loci in early development - and its functional consequences for replication dynamics - remain an important next step.

Another limitation concerns the molecular specificity and origin of the HSATII RNA involved in triplex formation. Multiple studies have shown that lncRNA-DNA triplex formation can occur in both cis and trans configurations (Mondal et al., 2015; O’Leary et al., 2015; Li et al., 2016). Within the framework of the presented hypothesis, such interactions could arise through local (cis) binding between Y-derived HSATII RNA and its own DNA, or through nonlocal (trans) pairing by RNAs transcribed from HSATII arrays on other chromosomes. Although Y-linked HSATII sequences show measurable divergence from their autosomal and X-linked counterparts, they still remain largely homologous (Logsdon and Eichler, 2023), thus retaining partial complementarity that could permit RNA-DNA triplex formation in the trans mechanism. Moreover, because RNA-DNA triplex formation is RNA concentration dependent (Postepska-Igielska et al., 2015), the exceptionally high abundance of HSATII RNAs, together with the observed adjacent localization of HSATII DNA and RNA in the human cell nucleus (Hall et al., 2017), could promote occasional lower-affinity interactions through the trans pathway.

Clarifying the relative contributions of these local and cross-chromosomal interactions would further refine the mechanistic understanding of the proposed model and its broader implications for repeat-associated chromosomal instability.

Finally, the hypothesis focuses explicitly on HSATII because Y-linked HSATII has an unusually high predicted triplex-forming propensity. Nonetheless, RNAs derived from other satellite tandem repeat classes have been shown to be distinctly upregulated in the preimplantation stages of early embryogenesis (Yandım and Karakülah, 2019) and could, in principle, contribute to chromosomal instability through related mechanisms.

It is important to mention here that one particularly relevant candidate is HSATIII, which is closely related to HSATII at the sequence level, as both repeat families derive from ATTCC-based pentameric motifs (Dobrynin et al., 2020). Classic *in situ* hybridization studies have shown that HSATII and HSATIII probes can partially cross-hybridize, an effect attributed to sequence similarity between the two repeat families (Tagarro et al., 1994). This homology raises the possibility that transcripts derived from one family could, in principle, participate in triplex formation at the other’s DNA arrays. Two mechanistic scenarios therefore emerge for future investigation: (i) elevated HSATIII RNA levels might contribute to triplex formation on HSATII DNA and reinforce the mechanism proposed in this article; and (ii) HSATII RNA might similarly form triplex structures on HSATIII arrays, depending on local sequence compatibility. Importantly, neither trans-family triplex formation nor the intrinsic triplex-forming propensity of HSATIII DNA has been experimentally assessed, and these possibilities therefore remain speculative. It is also notable that HSATIII transcription is strongly induced by activation of the heat-shock pathway (Jolly et al., 2004), although its expression dynamics in early human embryogenesis remain poorly characterized. Thus, if stressors that activate the heat-shock pathway were to occur during early development, the resulting increase in HSATIII RNA could potentially influence the proposed mechanism.

Taken together, comparative genome-wide analyses of triplex and other RNA-DNA structure formation propensities across multiple tandem repeat classes on different chromosomes, using standardized prediction parameters and experimental benchmarking, would help determine whether the proposed Y-linked HSATII associated phenomenon is distinctive or reflects a broader category of repeat-associated genomic vulnerability in early embryogenesis.

### Proving the hypothesis

Testing the proposed hypothesis will require an integrative approach combining biochemical, cellular, and genomic assays to evaluate whether HSATII RNA can participate in triplex formation at Y-linked HSATII loci and whether such structures contribute to replication stress and chromosome missegregation - a potential source of LoY.

A critical first step is to establish whether endogenous RNA-DNA triplexes form at HSATII loci in early embryonic contexts and whether their distribution reflects the bioinformatic prediction that Y-linked HSATII is exceptionally triplex-prone (Hegyi and Lexa, 2024). This could be addressed with Triplex-Seq, which combines RNase H digestion to eliminate interference from RNA-DNA heteroduplexes, such as those present in R-loops (Cerritelli and Crouch, 2009), followed by psoralen crosslinking to stabilize triplex structures and subsequent immunoprecipitation using anti-dsDNA or S9.6 antibodies (Leisegang et al., 2024). Finally, sequencing of the isolated RNA and DNA could be used to generate genome-wide HSATII-associated triplex maps (Sentürk Cetin et al., 2019; Leisegang et al., 2022). In parallel with this genome-wide approach, more targeted assays could help validate the intrinsic triplex-forming capacity of specific HSATII arrays. Locus-specific triplex capture assays employing biotinylated probes, with or without prior psoralen crosslinking to stabilize triplex structures, can test the intrinsic triplex-forming propensity of different HSATII arrays (Postepska-Igielska et al., 2015; Alfeghaly et al., 2021). However, these latter techniques primarily assess sequence-based triplex potential rather than endogenous RNA-driven triplex formation. Together, these strategies could provide valuable supporting evidence for a Y-linked bias in HSATII triplex formation.

The next step is to determine whether triplex formation at Y-linked HSATII arrays can cause replication fork stalling, and whether this, in turn, may lead to chromosomal instability and subsequent Y loss (LoY). To test this, assays that couple replication dynamics with targeted perturbations are essential. Replication fork progression at HSATII arrays can be evaluated using DNA fiber analysis (Quinet et al., 2017; Halliwell et al., 2020) or single-molecule analysis of replicated DNA (SMARD) (Kaushal and Freudenreich, 2019), both of which visualize fork speed and detect stalling at specific genomic regions.

To establish whether replication fork stalling at HSATII loci is mediated by RNA-DNA triplexes, several complementary strategies could be applied. One approach is to manipulate HSATII RNA abundance in early embryonic cells, either through antisense oligonucleotide (ASO)/LNA-gapmer mediated silencing (Amodio et al., 2018; Lellahi et al., 2018) or through vector-mediated overexpression (Gupta et al., 2010; Landers et al., 2021). Subsequent DNA fiber analyses or SMARD would then test whether changes in HSATII RNA levels alter replication fork progression. If such effects were observed, this would suggest that HSATII RNA contributes to replication stress beyond the intrinsic non-B-DNA conformations of HSATII repeats, although the precise structural mechanism would still require clarification. To probe this mechanism more directly, RNase H digestion could be adapted from Triplex-Seq protocols (Sentürk Cetin et al., 2019), eliminating RNA-DNA heteroduplexes such as R-loops while leaving triplexes intact. The persistence of fork stalling after R-loop removal would implicate triplex structures more directly. Similarly, depletion of helicases known to participate in triplex resolution: DHX9 (Jain et al., 2010) WRN and BLM (Brosh et al., 2001), or FANCJ/BRIP1 (Sommers et al., 2009), followed by replication assays could reveal increased fork stress consistent with a triplex contribution, although these enzymes also act on other secondary DNA structures. Finally, the pharmacological stabilization of triplexes with coralyne, an alkaloid which has been shown to promote triplex formation *in vitro* (Biver et al., 2010), could further strengthen causal evidence if it increases replication fork stress specifically at HSATII loci.

To determine whether HSATII RNA-derived triplex formation on the Y chromosome may cause biased chromosomal instability resulting in a predisposition for Y chromosome loss, cytogenetic analyses are needed. Because TS occurs in roughly one in 2,500 live female births (Ibarra-Ramírez et al., 2023), emergence of the LoY phenotype probably requires a combination of cellular predispositions, which might be derived partially from the proposed mechanism, and several variable factors, as underlined before in the *Limitations of the hypothesis* section. Consequently, spontaneous Y loss events - as with chromosomal missegregation in general - are likely too infrequent in unperturbed early embryonic cells to be efficiently captured in experimental settings (Thompson and Compton, 2008). To address this methodological limitation, mild, nonspecific replication stress could be introduced using agents such as aphidicolin or hydroxyurea at sublethal concentrations (Wilhelm et al., 2019). These compounds act through well-established mechanisms: transient slowing of DNA polymerase activity, depletion of nucleotide pools, and modest perturbation of spindle dynamics (Courbet et al., 2008; Kotsantis et al., 2016), thereby increasing the baseline level of chromosomal stress across the genome without favoring any specific pathway or chromosome. This in turn is essential to avoid interference with the triplex-associated mechanism being tested. When applied in this controlled manner, aphidicolin or hydroxyurea would simply facilitate the observation of relatively rare events within a tractable timeframe, enabling more efficient validation of the proposed hypothesis without altering its underlying mechanism.

Following the adjustment described above, several complementary assays could be employed to monitor chromosomal outcomes. Immunofluorescence staining for markers of ultrafine bridges (UFBs), such as PICH or BLM (Chan et al., 2007; Bizard et al., 2018) as well as DAPI-based visualization of bulky chromatin bridges during mitosis (Siri et al., 2021), would enable quantification of segregation stress. Micronucleus assays could also be used to detect lagging or missegregated chromosomes (Catalán et al., 1998). Combining these approaches with Y chromosome-specific fluorescence *in situ* hybridization (FISH) (Stewart et al., 2008; Dumont et al., 2020), would allow determination of whether chromosome loss events display a Y-linked bias during early embryogenesis. An increased frequency of Y-specific segregation defects - particularly under conditions of experimentally elevated HSATII RNA expression, stabilized triplex structures, or impaired triplex resolution pathways - would provide compelling evidence that HSATII RNA-mediated triplex formation contributes to Y chromosome instability in early embryogenesis. These findings would not only deepen the understanding of TS etiopathology but also emphasize the broader relevance of repeat-associated RNA-DNA interactions in human chromosomal stability.

## Discussion

Experimental evaluation of the proposed hypothesis has the potential to reveal a previously unrecognized molecular basis for Y chromosome instability during the preimplantation stages of early human embryogenesis - a process that, in combination with other modifying factors, could contribute to the TS phenotype.

If validated, this mechanism would not only expand the understanding of TS etiopathology but also reopen the discussion of the mechanistic background of chromosomal disorders traditionally regarded as stochastic. Confirming an RNA-DNA triplex structure based contribution to Y chromosome loss within the early embryonic molecular window of transiently elevated HSATII RNA levels would represent a conceptual shift, linking what was once viewed as random segregation error to a definable, potentially traceable molecular process.

A key implication of this hypothesis lies in its potential to guide the search for individual molecular risk factors for Y chromosome instability in early embryogenesis. If the triplex-mediated mechanism contributes to Y loss, susceptibility may vary according to both sequence features and the efficiency of the molecular pathways that resolve such structures. Because satellite DNA, including HSATII, evolves rapidly and exhibits considerable interindividual variation (Miga, 2019; Logsdon et al., 2024) comparative analyses of Y-linked HSATII arrays across diverse male genomes and, where feasible, within mosaic Turner individuals retaining Y chromosomal fragments, could identify sequence variants associated with elevated triplex-forming potential. The incorporation of recently developed methods that enable efficient sequencing of highly repetitive regions would make such analyses increasingly feasible (Altemose et al., 2022). Moreover, the variability in the enzymatic systems responsible for resolving triplex and other secondary DNA structures, as outlined in the *Proving the hypothesis* section, could further influence how efficiently replication stress arising from the proposed mechanism is mitigated. Given that oocyte-derived cytoplasmic factors provide the molecular environment for the earliest embryonic divisions (Mitchell, 2022; Jentoft et al., 2023), maternal differences in these repair pathways may be particularly relevant. In the longer term, identification of such interindividual variables could enable risk stratification for Y chromosome instability in potential embryos and provide a framework for understanding why TS associated karyotypes arise only in a subset of embryos despite the general presence of HSATII RNA during early human development.

An additional consideration concerns the potential relevance of HSATII transcription dynamics during male gametogenesis. While HSATII upregulation during oocyte maturation is well documented, comparable data for the male germ line are not currently available. Understanding whether HSATII transcripts are similarly regulated in spermatogenesis would help determine if the proposed mechanism of HSATII-RNA–mediated Y chromosome instability might operate not only in early embryogenesis, but also during male gamete production. Although speculative at present, this possibility highlights the value of extending HSATII expression profiling to human spermatogenic stages in future studies.

Another implication arises when this model is considered alongside the observation that HSATII transcription is strongly inducible by cellular stress (Smurova and De Wulf, 2018; Fonseca-Carvalho et al., 2024). Although such activation has been described primarily in somatic systems, it is plausible that early embryonic cells, already characterized by high baseline HSATII RNA expression, could exhibit even further upregulation under stress conditions. Notably, these cells also possess inherently limited cell-cycle checkpoint control (Brantley and Di Talia, 2021; Horakova et al., 2024), which may render them even less capable of mitigating the consequences of HSATII RNA-driven chromosomal instability compared with differentiated somatic cells. This underscores the importance of examining factors that modulate HSATII RNA abundance during early development, including environmental, metabolic, or stress-related influences, as such variables may act as upstream modifiers of Y chromosome stability within the proposed framework.

From a broader perspective, the proposed model highlights the potential importance of pericentromeric satellite biology in chromosomal disorder mechanisms. This hypothesis may therefore contribute to the growing awareness that tandemly repeated sequences constitute an active regulatory layer of genome maintenance which modulation may have far-reaching implications for human disease and evolutionary biology.

## Conclusion

TS etiopathology is currently regarded as a largely stochastic process involving sex chromosome loss or structural alteration, and cases arising from Y chromosome loss lack a clear mechanistic explanation. The presented hypothesis seeks to address this gap by proposing that RNA-DNA triplex formation involving the HSATII RNA rich molecular window and Y-linked HSATII arrays could represent a previously unrecognized contributor to Y chromosome instability during early embryogenesis. This framework provides a potential molecular basis for a subset of TS cases attributable to Y chromosome loss and situates this phenomenon within the broader emerging view that pericentromeric satellite sequences play active roles in genome stability.

Although experimental validation is needed before any definitive conclusions can be drawn, confirmation of this mechanism would not only contribute to clarifying one of the least understood aspects of TS but also broaden the understanding of how tandem repeat-associated RNA-DNA interactions influence chromosomal stability in human development. Importantly, it could prompt renewed investigations into modifying factors: genetic, molecular, or environmental, that determine why such instability manifests only in certain embryos. In the longer term, this line of inquiry may establish a conceptual and methodological foundation for assessing Y chromosome instability risk in potential embryos based on parental molecular profiles, marking a shift from viewing chromosomal loss as purely random toward recognizing it as a partially predictable, biologically governed process.

## Data Availability

The original contributions presented in the study are included in the article/supplementary material, further inquiries can be directed to the corresponding author.

## Abbreviations

TS, Turner syndrome; LoY, Loss of Y chromosome; CENP-B, Centromere protein B (major centromere autoantigen B); HSATII, Human satellite II; lncRNA, Long non-coding RNA; DUX4, Double homeobox 4; ATM, ATM serine/threonine kinase (Ataxia-telangiectasia mutated); IE1, Immidiate early protein 1 (viral HCMV protein); IE2, Immidiate early protien 2 (viral HCMV protein); HSATIII, Human satellite III; PATO, *high-PerformAnce TriplexatOr* (triplex prediction algorithm); Hi-C, High-throughput chromosome conformation capture; urDNA, Under-replicated DNA; UFBs, Ultrafine DNA bridges; ASO, Antisense oligonucleotide; LNA-gapmer, Locked nucleic acid-gapmer; DHX9, DEAH-box helicase 9 (RNA helicase A); WRN, Werner syndrome RecQ-like helicase; BLM, Bloom syndrome RecQ-like helicase; FANCJ/BRIP1, BRCA1-interacting protein C-terminal helicase 1; SMARD; Single-molecule analysis of replicated DNA; PICH, Plk1-interacting checkpoint helicase; DAPI, 4′,6-Diamidino-2-phenylindole; FISH, Fluorescence *in situ* hybridization.
